# Utility and Challenges of Imaging in Peripheral Vestibular Disorder Diagnosis: A Narrative Review

**DOI:** 10.3390/diagnostics15101272

**Published:** 2025-05-16

**Authors:** Gabriela Cornelia Musat, Codrut Sarafoleanu, Mihai Alexandru Preda, Calin Petru Tataru, George G. Mitroi, Andreea Alexandra Mihaela Musat, Mihnea Radu, Ovidiu Musat

**Affiliations:** 1 ENT Department, Faculty of Dentistry, “Carol Davila” University of Medicine and Pharmacy, 020021 Bucharest, Romania; gabriela.musat@umfcd.ro (G.C.M.); codrut.sarafoleanu@umfcd.ro (C.S.); mihai.preda@umfcd.ro (M.A.P.); 2 Department of Ophthalmology, Faculty of Medicine, “Carol Davila” University of Medicine and Pharmacy, 020021 Bucharest, Romania; calin.tataru@umfcd.ro (C.P.T.); ovidiu.musat@yahoo.com (O.M.); 3 Department of Dermatology, Faculty of Medicine, University of Medicine and Pharmacy of Craiova, 200349 Craiova, Romania; 4 Doctoral School, “Carol Davila” University of Medicine and Pharmacy, 020021 Bucharest, Romania; andreea-alexandra.musat@rez.umfcd.ro; 5 Department of General Surgery, Clinical Hospital Colentina, 020125 Bucharest, Romania; mihnea.radu96@yahoo.com

**Keywords:** vestibular disorders, vertigo, dizziness, computed tomography, magnetic resonance imaging, Meniere’s disease, vestibular neuritis, acoustic neuroma, superior canal dehiscence, benign paroxysmal positional vertigo

## Abstract

This review focuses on the contribution of medical imaging in the diagnosis of peripheral vestibular disorders. This is a narrative review based on a focused literature search conducted using PubMed and the Cochrane Library. Imaging is not usually recommended in initial consultations for vestibular disorders because only 5–10% of MRI scans reveal findings directly related to the disease. The study is a review of the literature that highlights the utility and limitations of imaging such as computed tomography (CT) and magnetic resonance imaging (MRI). It follows the diagnostic approach from history and physical examination to laboratory tests and imaging. Some conditions like vestibular neuritis and benign paroxysmal positional vertigo (BPPV) have limited imaging utility due to the fine details required. Conversely, high-resolution CT and MRI are important for diagnosing Meniere’s disease, acoustic neuroma, and superior canal dehiscence. The role of imaging varies a lot among specific conditions. Advances in imaging technology, particularly high-resolution MRI, promise enhanced diagnostic capabilities.

## 1. Introduction

Vertigo and dizziness are among the most frequently encountered symptoms in clinical practice, presenting a diagnostic challenge across multiple specialties, including primary care, neurology, and otolaryngology. Vertigo may arise from either peripheral vestibular disorders—such as BPPV, vestibular neuritis, or Meniere’s disease—or central causes like stroke and multiple sclerosis. Distinguishing between these origins is critical, as their diagnostic approach and clinical management differ significantly. Epidemiological data indicate that vertigo and dizziness affect between 15% and 20% of adults annually [1], with a broader prevalence of up to 56% in some populations [2], and an increasing incidence correlated with advancing age [3]. These symptoms are most commonly attributed to dysfunction within the peripheral vestibular system [4], although central nervous system etiologies must also be considered [5]. Diagnosing the exact site and nature of these lesions involves a thorough history, an attentive clinical examination, functional vestibular testing, and a variety of imaging modalities—including CT, MRI, and, in selected cases, ultrasound or functional neuroimaging techniques such as PET or functional MRI [6].

The diagnostic evaluation of vestibular symptoms is multifactorial, typically beginning with a comprehensive clinical history and physical examination. Skilled clinicians are capable of reaching a probable diagnosis in approximately two-thirds of cases based solely on anamnesis and symptomatology [6,7,8,9]. Bedside assessments, including the evaluation of vestibulo-ocular and vestibulo-spinal reflexes [10,11,12], alongside observation of spontaneous or provoked nystagmus [13,14], further assist in localizing the lesion. Functional vestibular tests such as video-oculography, caloric testing, the video head impulse test (vHIT), vestibular evoked myogenic potentials (VEMP), and posturography are frequently utilized to support the clinical hypothesis and characterize the extent of dysfunction [15,16].

Despite the central role of clinical assessment, medical imaging has become an essential adjunct in selected cases. The most commonly used imaging modalities are computed tomography (CT) and magnetic resonance imaging (MRI). CT and MRI are commonly used to evaluate vestibular and CNS structures, each with unique strengths in assessing bone and soft tissue, respectively. MRI with contrast can detect pathologies such as vestibular schwannomas, ischemic lesions, labyrinthitis, and endolymphatic hydrops, especially with advanced techniques such as delayed gadolinium-enhanced 3D-FLAIR sequences [14,15,16,17,18].

While imaging is not routinely recommended for every patient with vertigo, it is essential in cases presenting with atypical features, neurological signs, or unilateral progressive hearing loss or when central causes such as stroke or tumor are suspected. Nevertheless, imaging is often overused. Approximately 50% of scans are considered unnecessary in general medical practice [17], and only 5–10% of MRIs for vestibular symptoms reveal findings directly related to the underlying pathology [18]. Inadequate imaging interpretation and over-reliance on imaging in lieu of clinical evaluation may lead to misdiagnosis or delayed diagnosis, especially in conditions such as posterior circulation stroke, where early MRI can still miss up to 20% of lesions [16,17,18].

Given these diagnostic challenges, imaging has both utility and limitations. Misdiagnosis of peripheral vestibular disorders—by either missing a serious central cause or attributing central symptoms to a benign peripheral condition—can have serious consequences, including delayed treatment, inappropriate interventions, and increased healthcare costs. Therefore, the use of imaging must be judicious, integrated into the broader clinical context, and guided by evidence-based criteria.

This review aims to critically evaluate the role of imaging in the diagnosis of peripheral vestibular disorders, examining its diagnostic accuracy, indications, limitations, and emerging techniques, such as ultrasound, functional MRI, and artificial intelligence applications. In doing so, we also highlight the significance of imaging in specific disorders such as vestibular neuritis, Meniere’s disease, benign paroxysmal positional vertigo (BPPV), superior canal dehiscence, and acoustic neuroma and propose a framework for appropriate imaging use in clinical practice.

## 2. Materials and Methods

This review is narrative in nature and does not follow PRISMA or formal systematic review criteria, although it employs a focused literature search strategy.

A literature search was conducted using PubMed and the Cochrane Library from January 1986 to February 2025. The Boolean string used was ((“vestibular disorders”) AND (“MRI”) AND (“CT”) AND (“diagnosis”)). We also manually screened the reference lists of relevant articles to identify additional sources. The search was limited to studies published in English. No formal MeSH terms or filters were applied due to the narrative nature of the review, but preference was given to peer-reviewed studies, systematic reviews, and high-impact clinical research. The search encompassed studies published between January 1986 and February 2025. A total of 163 potentially relevant publications were identified, consisting of 159 from PubMed and 4 from the Cochrane Library. Given the clinical focus and the predominance of relevant studies in PubMed and Cochrane, these databases were deemed sufficient for the scope of this narrative review.

Included sources comprised narrative reviews, systematic reviews, clinical studies, and case reports relevant to imaging in vestibular disorders. Publications such as letters to editors, conference abstracts, editorials, opinion articles, and unpublished materials were explicitly excluded to maintain the scientific rigor and clinical relevance of the review. Only studies published in English were considered for inclusion in this review.

After an initial title screening, the retrieved articles underwent a structured review process. The authors evaluated the relevance and clinical applicability of studies based on topic relevance and publication quality.

**Figure 1:**
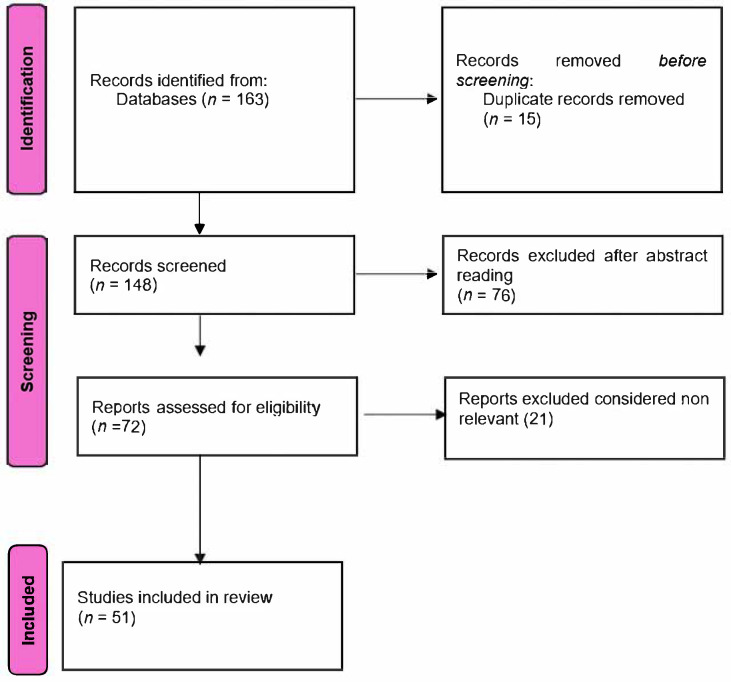
Flowchart of the literature search and article selection process.

Figure 1 provides a general overview of the literature search and selection process used in this narrative review. This paper presents a narrative review. While it uses systematic search terms to guide article inclusion, it does not follow formal PRISMA or systematic review methodology, as the goal was to provide a thematic synthesis rather than exhaustive systematic evaluation. Some of the articles included in the study were not available in full text-open access.

## 3. Imaging Modalities in Vestibular Disorder Diagnosis

There are imaging tools available for evaluating patients with vestibular disorders. To accurately diagnose vestibular disorders, a detailed visualization of the inner ear, the trajectory of the vestibulo-cochlear nerves, and central nervous system pathways can be useful [19]. Taking into account that most of the vestibular disorders originate from the peripheral vestibular system, a detailed image of the vestibular end organs is required. The size of the inner ear and of the vestibular structures involved in maintaining balance necessitates an imaging modality able to visualize small-scale details. The imaging modalities usually used in the assessment of vestibular disorders are computed tomography and MRI. In present, none of these methods provide enough information about minute details of the anatomy of the vestibular end organs in order to assess their modifications in different vestibular disorders because of the fact that the resolution is insufficient. Physicians usually refer patients for imaging modalities to diagnose a possible central vestibular disorder. A progressive hearing loss associated with vestibular symptoms is a good indication for a radiologic evaluation because it can indicate an acoustic neuroma. A clinician should consider imaging modalities whenever the patient has neurologic symptoms, cerebrovascular disease risk factors, or unilateral progressive hearing loss [20].

Computed tomography is an advanced radiological technique that employs ionizing X-rays and computer-based reconstruction to generate high-resolution cross-sectional images based on the differential attenuation of tissues. In the evaluation of vestibular disorders, temporal bone CT is particularly effective for evaluating bony structures and the otic capsule. It is commonly used to detect structural anomalies such as labyrinthine ossification, superior semicircular canal dehiscence, congenital malformations, fractures, or postoperative changes like prosthetic displacement. Due to its excellent spatial resolution, CT remains the modality of choice when assessment of osseous pathology is required. However, its inability to accurately visualize soft tissue limits its diagnostic value in detecting subtle inner ear pathology, including inflammatory or hydropic changes. In acute settings, CT is frequently employed to exclude intracranial hemorrhage or skull base trauma. Nonetheless, the diagnostic yield of CT and CT angiography in patients presenting with isolated vertigo remains relatively low, and its use should be reserved for specific clinical scenarios where central or structural bony lesions are suspected [21]. New data suggest the possibility of identifying the side of the vestibular deficit using head CT to evaluate a sign called the “vestibular eye sign” (VES), which is defined as an eye deviation that correlates with the slow phase of the nystagmus [22].

MRI is a non-invasive diagnostic technique that utilizes strong magnetic fields and radiofrequency pulses to generate detailed anatomical images based on tissue-specific signal characteristics. Unlike CT, MRI does not expose the patient to ionizing radiation, making it a safer modality for repeated imaging, especially in younger or vulnerable populations. Its superior soft tissue contrast allows for precise evaluation of the membranous labyrinth, internal auditory canal, cerebellopontine angle, and associated neurovascular structures. Cranial MRI is the preferred modality for assessing brain lesions. MRI is particularly valuable in identifying soft tissue lesions such as vestibular schwannomas, inflammatory changes, and endolymphatic hydrops when enhanced with gadolinium-based contrast agents. Additionally, diffusion-weighted imaging (DWI) enables early detection of ischemic events in the posterior fossa. Despite these advantages, the resolution of conventional MRI may still be insufficient to visualize minute abnormalities within the vestibular end organs, limiting its sensitivity in certain peripheral vestibular disorders. Therefore, MRI is primarily reserved for cases with atypical presentations, central nervous system involvement, or progressive unilateral auditory symptoms [23,24]. MRI is required to image the structures of the inner ear with the perilymphatic and the endolymphatic spaces and the vestibular nerves. Although MRI with contrast agents like gadolinium and the special sequences provide better visualization of the inner ear and vestibular nerve, it often lacks sufficient detail for comprehensive assessment. MRI is the imaging modality of choice for identifying brain lesions and is essential in differentiating peripheral from central vestibular disorders, including stroke and space-occupying lesions [25]. DWI is used for the assessment of routine ischemia, while gadolinium is used for characterizing inflammatory and tumoral lesions. Nonetheless, both MRI and CT are instrumental in distinguishing peripheral from central vestibular disorders [18].

### 3.1. Specific Vestibular Disorders and Imaging Utility

#### 3.1.1. Vestibular Neuritis

Vestibular neuritis is a peripheral vestibular disorder considered to be caused by an inflammation of the vestibular portion of the eighth nerve presenting with vertigo, nausea, and gait imbalance. It is also known as vestibular neuronitis. The Barany Society suggests renaming it “acute unilateral vestibulopathy” [26]. This peripheral vestibular disorder involves damage to the vestibular nerve fibers, possibly due to a viral infection, although a vascular etiology cannot be totally rejected. Nowadays, the viral theory is accepted, and a reactivation of the HSV-1 latent in the vestibular ganglia is incriminated [27]. The annual incidence is reported as 3.5–15.5 for 100,000 persons [28]. It manifests as an acute vestibular syndrome due to sudden loss of vestibular function without auditive symptoms. Symptoms include acute vertigo, imbalance, nausea, and vomiting, typically resolving slowly with central compensation. Symptoms are accentuated, not triggered by head movement. Usually, it lasts for several days, but it can last weeks to months until the complete resolution of symptoms. At the clinical exam, patients exhibit horizontal or horizontal-rotatory nystagmus toward the unaffected ear and deviation of the body toward the diseased side. The nystagmus is always unidirectional with the slow phase toward the diseased ear and follows the law of Alexander (it is more intense when the patient is looking to the healthy side). The head impulse test (HIT) is positive on the lesioned side. Video HIT, VNG with calorics, and VEMP tests show deficits on the affected side and are capable of detecting which division of the vestibular nerve is involved. Auditory function is usually preserved. Imaging has limited utility in diagnosing vestibular neuritis, as MRI and CT generally lack the resolution to detect such fine details. The diagnosis of vestibular neuritis is based on anamnesis and physical examination. Contrast-enhanced MRI can sometimes show signal enhancement in the vestibular nerve [29,30]. In cases where vestibular neuritis is suspected but the diagnosis remains uncertain, contrast-enhanced MRI may be valuable in detecting signal enhancement or swelling in the affected vestibular nerve. Studies using delayed gadolinium-enhanced 3D FLAIR sequences have demonstrated visible abnormalities in the vestibular nerve, suggesting possible inflammation or breakdown of the blood–nerve barrier. These findings support the diagnostic use of MRI not only for exclusion of central causes but also for direct visualization of peripheral nerve involvement [31].

#### 3.1.2. Meniere’s Disease

It is also known as idiopathic endolymphatic hydrops because the presumed pathophysiologic mechanism is increased pressure in the endolymphatic system. Although the disease was described by Dr Prosper Meniere in 1861 [32], controversies regarding the pathophysiology still exist and the disease has unclear pathophysiology [33]. Meniere’s disease is idiopathic by definition, but Meniere’s syndrome occurs secondary to various conditions that interfere with the production or resorption of endolymph such as autoimmune disfunction, endocrine abnormalities, electrolyte imbalance, and medication. Meniere’s disease is characterized by spontaneous vertigo attacks (lasting 20 min to 24 h) accompanied by nausea, vomiting, fluctuating hearing loss, tinnitus, and a sensation of ear pressure. It affects 20 to 200 per 100,000 persons per year, most frequently between 40 and 60 years of age [34]. The prevalence of the diseases varies widely from 15 per 100,000 in US to 157 per 10,000 in UK [2]. There are different guidelines for the diagnosis of the disease issued by different medical societies. The American Society of Otolaryngology and Head and Neck Surgery bases the diagnosis of Meniere’s disease solely on clinical symptoms [35]. According to the guideline published in 2020, Meniere’s disease is a clinical condition defined by spontaneous attacks of vertigo (lasting from 20 min to 12 h), documented sensorineural hearing loss (before, during, or after the attack), and other fluctuating aural symptoms such as tinnitus or ear fulness. The physical examination findings are not remarkable or specific to the disease because the status may vary depending on the phase. Audiometric testing may show low to mid frequencies hearing loss [36]. Different electrophysiological tests have been used as diagnostic modalities for the disease, but none of them reached international consensus. The electrophysiologic tests or other functional tests that have been proposed as a diagnostic procedure are electrocochleography (ECoG), VNG, VEMP, or otoacoustic emissions, but none of them is pathognomonic [37]. ECoG measures the ratio between the summation potential (possibly produced by the displacement of the basilar membrane) and the nerve action potential, with hydrops being suggested by an elevation of this ratio bigger than 35%. Videonystagmography records and measures the nystagmus, but the direction may vary during or after an attack and it is not a reliable indicator of the diseased side. In general, the nystagmus has a fast phase away from the affected ear because Meniere’s diminishes the reactions of the diseased ear; however, an irritative phase may appear during the attack.

**Figure 2:**
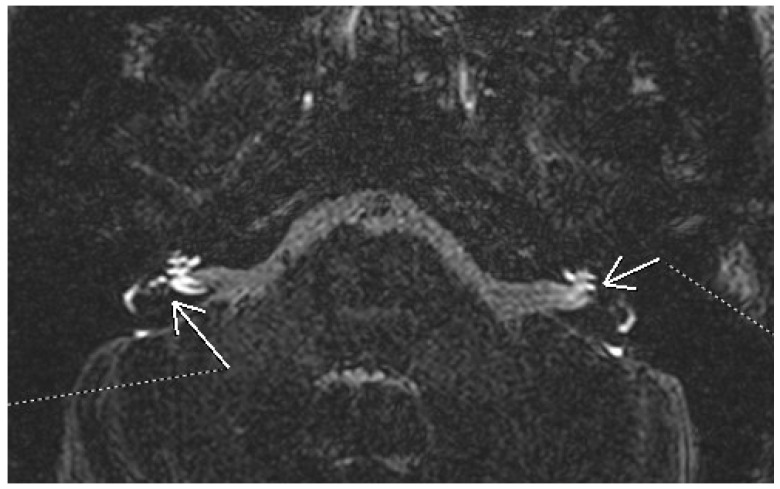
3D FLAIR MRI using a 3T scanner and delayed gadolinium protocol, showing bilateral cochlear and vestibular hydrops.

The guidelines of the AAO-HNS state that physicians may offer MRI of the internal auditory canal (IAC) and posterior fossa as an option in patients with possible Meniere’s disease and audiometrically verified asymmetric hearing loss [35]. Imaging, particularly high-resolution CT and MRI, has a controversial utility [38]. CT may show some modifications compatible with Meniere’s disease. A narrowed vestibular aqueduct is encountered more frequently in affected ears [39,40]. Non-contrast enhanced MRI can show in advanced cases elongation of the saccule, diminished fluid in the cochlear aqueduct, or invisibility of the endolymphatic sac and duct [41,42]. A key advancement in diagnosing endolymphatic hydrops has been the use of contrast-enhanced MRI with gadolinium, which enables visualization of inner ear fluid compartments. Initially, it was administered intratympanically with delayed acquisition. An example of bilateral endolymphatic hydrops using 3D FLAIR MRI after gadolinium administration is shown in Figure 2. The 3D-FLAIR MRI images were obtained using a 3T scanner (Siemens Magnetom Lumina, Siemens Healthcare S.R.L., Bucharest, Romania) with a delayed acquisition protocol approximately 4 h after intravenous administration of gadolinium-based contrast agent. Parameters typically include slice thickness of 0.5–1.0 mm, high in-plane resolution, and signal nulling to distinguish endolymphatic from perilymphatic spaces. Later it was reported that endolymphatic hydrops can be visualized also after intravenous administration of contrast [43]. The gadolinium based contrast agent accumulates in the perilymphatic space, allowing the visualization of the less permeable endolymphatic space. In 3D FLAIR MRI, this space appears as signal voids surrounding hyperintense perilymph, so it highlights changes in the inner ear consistent with endolymphatic hydrops. These changes can be enlargement of the cochlear endolymphatic space, an increment of the vestibular endolymphatic space occupying over one-third of the vestibular space, and a saccule larger than the utricle [44,45]. Different qualitative and semiquantitative criteria for describing endolymphatic hydrops have been proposed over the years. The most well-known is the Nakashima criteria. It defines vestibular hydrops as an endolymph/perilymph ratio of >33%. For the cochlear hydrops, any visual hydrops of the cochlear duct is considered hydropic [46]. Barath later modified this classification into a three-point scale categorizing the cochlear and vestibular hydrops as none, grade 1, and grade 2 [47]. In addition to hydrops, there are other MRI features compatible with Meniere’s. Non-visualization of the saccule has been described in some patients. The proposed explanation is either the collapse or the fistulation noticed in some histopathologic studies [48]. Recently, a “round window sign” has been described as a hyperintense signal in the region of the round window on delayed 3D FLAIR. This has been hypothesized as a sign of perilymphatic fistula [49]. Further research is needed to establish the role of MRI in the diagnosis of Meniere’s disease to improve and validate the techniques in acquisition and interpretation.

One of the critical roles of imaging in the evaluation of suspected Meniere’s disease is to exclude secondary causes of endolymphatic hydrops, often referred to as secondary perilymphatic hydrops. These cases may present with clinical features mimicking idiopathic Meniere’s disease but occur in association with other identifiable etiologies such as head trauma, inner ear surgery, chronic otitis media, autoimmune inner ear disease, or congenital anomalies. In such scenarios, the endolymphatic hydrops is not idiopathic but rather a manifestation of an underlying condition that alters the homeostasis of inner ear fluids. Distinguishing primary from secondary hydrops is essential for both prognosis and therapeutic planning. Imaging, especially MRI with delayed gadolinium enhancement, plays a supportive role in this differentiation by helping identify structural abnormalities, labyrinthine fibrosis, perilymphatic fistulas, or prior surgical changes. While imaging alone may not definitively establish etiology, it provides critical clues that guide further investigation. Thus, in addition to its diagnostic utility in visualizing endolymphatic hydrops, MRI contributes significantly to ruling out secondary causes, reinforcing the importance of a comprehensive diagnostic approach in patients with suspected Meniere’s disease [50]. High-resolution 3D-FLAIR MRI sequences have gained traction in clinical practice for diagnosing endolymphatic hydrops, particularly in specialized neurotology centers. These protocols are now incorporated in tertiary care settings for the evaluation of Meniere’s disease, as supported by recent clinical guidelines [51].

#### 3.1.3. Benign Paroxysmal Positional Vertigo (BPPV)

Imaging studies are not typically indicated in the diagnosis of BPPV, as the disorder results from microscopic dislodged otoconia that are not visible with current imaging techniques. CT and MRI offer no diagnostic benefit unless atypical features raise concern for alternative diagnoses, in which case imaging serves primarily to rule out other structural or central pathologies. This highlights a key limitation in the applicability of imaging for certain peripheral vestibular conditions [52,53,54,55,56].

#### 3.1.4. Acoustic Neuroma

This benign tumor develops from the Schwann cell sheath of the eighth nerve in the internal auditory canal invading the cerebellopontine angle in its growth. The WHO classifies schwannomas as grade I benign tumors. It seems that most of the time it develops from the inferior division of the vestibular component of the nerve. Usually, it is a unilateral sporadic tumor. Bilateral vestibular schwannomas are suggestive of type 2 neurofibromatosis, a genetic autosomal dominant disorder [57]. From a histopathologic point of view, the schwannomas are classified as classic schwannomas (with 2 distinct regions Antoni A and B), cellular schwannomas (relatively uncommon), plexiform schwannomas (associated with schwanomatosis, NF2 and other syndromes), and melanotic schwannomas (potentially malignant neoplasm). The incidence of vestibular schwannomas is 1.2 cases per 100,000 persons/year [58]. It accounts for 6–8% of intracranial tumors and 80% of cerebello-pontine angle tumors. Schwannomas may exist for years without any sign because these tumors exhibit slow growth. The tumors often cause progressive hearing loss and nonpulsatile tinnitus. The vestibular symptoms are less pronounced due to gradual tumor growth and central compensation [59]. Neurologic symptoms may appear when the tumor is large and invades the cerebello-pontine angle and presses on the CNS structures. Facial nerve palsy is rare as a first presentation sign. It is a benign tumor, but it can be dangerous on account of the extension and the compression at the level of the adjacent structures [60,61]. Assessing the location, size, and growth rate is mandatory for identifying and establishing the treatment approach. Most of the tumors have an intra-canalicular component enlarging the porus acusticus (“trumpeted internal acoustic canal”), and extracanalicular extension may result in an “ice cream cone “aspect. A minority of tumors are only extracanalicular. The Koos grading system is used in order to classify the size of the tumor with impact on the hearing preservation rate after surgery [62]. This is the reason why imaging is of paramount importance in the diagnosis of the neuroma. It can differentiate acoustic neuromas from other cerebellopontine tumors such as meningiomas, metastases, cholesteatomas, vascular lesions, epidermoid cysts, and exophytic gliomas [63]. Vestibular schwannomas are well-circumscribed, encapsulated tumors separated from the nerve fibers. Small tumors tend to be solid, while large ones may have cystic degeneration or hemorrhage. Typically, there are no calcifications [63].

For the radiologic evaluation of vestibular schwannomas, MRI and CT scans are considered. MR is the preferred technique used for characterization, therapeutic planning, and post therapeutic evaluation for acoustic neuromas. CT is used whenever MRI is contraindicated for different reasons. CT may reveal internal acoustic canal enlargement or bone erosion, with contrast enhancing the lesion. In some cases, enhancement can be underwhelming especially in large, cystic tumors [64]. The lesion is hard to see especially on account of the adjacent petrous bone artifacts. MRI, particularly with contrast, is the “gold standard” [65], showing the tumor [64] as hypointense or isointense on T1 and hyperintense on T2 images, with intense contrast enhancement [66]. In Figure 2, there is the image of a huge vestibular schwannoma seen on a T1 sequence with contrast invading the cerebello-pontine angle and compressing the CNS adjacent structures. A target sign (a T2 hyperintensity circumscribing a central hypointensity) is a specific sign for a peripheral nerve sheath tumor, but it is not specific for a schwannoma.

In the last period, due to the accessibility of the MRI, a large number of schwannomas are discovered. Many of these tumors do not necessitate surgery immediately or other treatment modalities, and a “wait and scan” attitude is adopted. MRI is indeed instrumental in assessing tumor characteristics, including size, location, and growth rate. The decision to proceed with surgery is a complex one, influenced by various factors such as tumor growth rate, patient symptoms, and overall health. Regular MRI follow-up is crucial to monitor tumor progression and determine the optimal timing for surgical intervention. MRI is essential in deciding the moment of the surgery and in monitoring patients postoperatively, especially in patients with cochlear implantation following the removal of the tumor [67].

**Figure 3:**
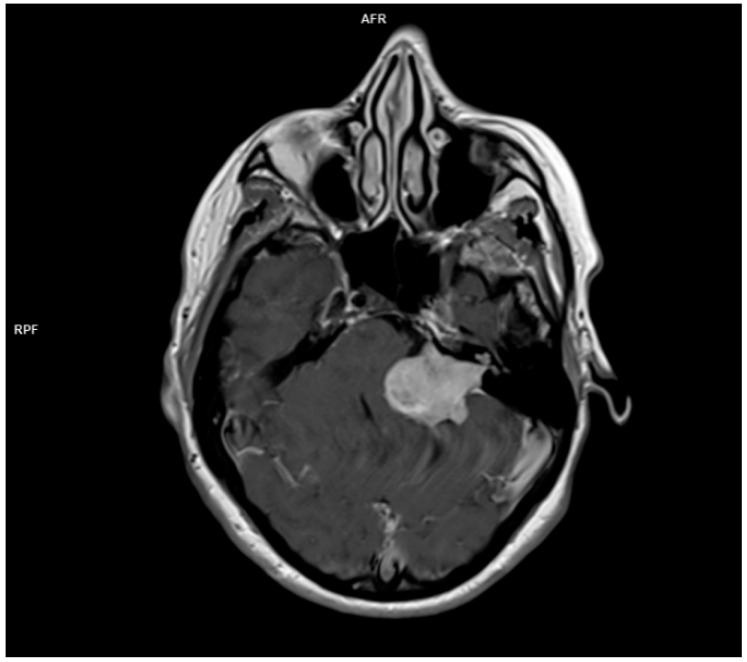
Axial T1-weighted contrast-enhanced MRI acquired at 3 Tesla (Siemens Magnetom Lumina, Siemens Healthcare S.R.L., Bucharest, Romania) with a 1 mm slice thickness. The image shows a large left vestibular schwannoma (acoustic neuroma) occupying the internal auditory canal and extending into the cerebellopontine angle, causing compression of the pons and left cerebellar hemisphere.

Another issue to discuss is the fact that one of the initial presentations of acoustic neuroma can be sudden sensory neural hearing loss (SSNHL) [68] or an acute vertigo mimicking a vestibular neuritis [69]. These atypical presentations underscore the importance of considering vestibular schwannoma in the differential diagnosis and prompt initiation of imaging studies, such as MRI, to rule out this potentially life-changing condition. Figure 3 illustrates a large vestibular schwannoma on a contrast-enhanced T1-weighted MRI, occupying the internal acoustic canal and extending into the cerebellopontine angle with compression of adjacent CNS structures. The tumor was imaged using a contrast-enhanced T1-weighted sequence (slice thickness 1 mm, 3T field strength) showing solid mass enhancement in the internal auditory canal extending into the cerebellopontine angle.

#### 3.1.5. Superior Canal Dehiscence

This is a rare disorder in which a bone defect creates a third window in the labyrinth [70]. The disease is characterized by vertigo triggered by loud sounds or pressure, hearing loss, autophonia, and tinnitus [71]. The vestibular symptoms induced by loud sounds are called the Tullio phenomenon because he was the first to describe it [72]. The vestibular symptoms induced by increased pressure in the external auditory canal are called Hennebert signs [73]. Minor found a series of patients with both Tullio and Hennebert signs and related the findings with an anatomic defect of the superior canal bone [74]. There is no single gold standard for the diagnosis of superior canal dehiscence; it is diagnosed using anamnesis, audiograms, VEMPs, and imaging [75,76]. The typical audiogram in superior canal dehiscence is a combination of increased air conduction thresholds with lowered bone conduction thresholds at low frequencies [77]. VEMPs are myogenic potentials collected either on the sternocleidomastoid muscle or the inferior oblique muscle as a response to the stimulation of the inner ear using loud sounds, galvanic stimulation, or a mechanical head tap. Although VEMPs evaluate the integrity of the entire reflex pathway, they are usually considered a test for the otolithic organs (saccule and utricle). Cervical vestibular evoked myogenic potentials (cVEMPs) evaluate the saccule and the integrity of its connection through the inferior vestibular nerve. Ocular vestibular evoked myogenic potentials (oVEMPs) measure the utricular and the superior nerve function. Consecutively to the apparition of a third window in the labyrinth, VEMP amplitudes increase, and thresholds decrease in case of superior canal dehiscence [78].

Imaging studies are critical for the diagnosis of superior canal dehiscence syndrome. High-resolution temporal bone CT scans with thin slices can detect bony defects in the superior semicircular canal at the level of arcuate eminence. If the slices are obtained at 0.5 mm instead of 1 mm, the accuracy is increased from 50% to 90% [70]. Although MRI is not traditionally used for the detection of superior canal dehiscence, recent research suggests its superior sensitivity and specificity, making it a valuable “rule-out” test. In a study published in 2013, MRI had a sensitivity of 96.5%, a positive predictive value of 61.1%, and a negative predictive value of 100% [79].

In Table 1, we present an overview of common vestibular disorders, highlighting key symptoms, diagnostic methods, and the role of imaging in their diagnosis. This table provides a concise summary of the information discussed.

#### 3.1.6. Differential Diagnosis with Central Vertigo

Vertigo is a common symptom in posterior circulation stroke. Although vertigo caused by a posterior circulation stroke is typically associated with other neurologic signs or symptoms, it is possible that a small infarct may manifest only as vertigo [80]. Acute stroke presenting only as vertigo can be easily mistaken as peripheral vestibulopathy. Roughly 25% of patients with acute vestibular syndrome have a potentially life-threatening disease, and stroke is encountered in 4–15% of cases [81]. Differential diagnosis between central and peripheral vertigo is critical in the case of acute vestibular syndrome. It is based on clinical history, targeted physical examination, and radiological evaluation.

The physical examination of the patient with acute vestibular syndrome is based on a battery of tests aimed to rapidly orientate the diagnosis in the emergency room. The HINTS battery of tests is used to assess the patient with acute vestibular syndrome in a bedside examination. It is composed of three different tests: HIT (head impulse test), N (nystagmus), and S (test of skew). HIT assesses how the eyes move in response to rapid movement of the head. In the case of a positive test, it is not possible to keep the eyes on the target, and a corrective saccade is noticed. The direction of the nystagmus can change in relation to the position of the eyes in cases of central pathology. The misalignment of the eyes is called skew deviation and is typical of central vestibular disorders [82]. The combination of the three signs, including positive HIT, direction-changing nystagmus, and the presence of a skew deviation, is indicative of a central vestibular disorder. Temporal bone CT is widely used in the emergency room for the evaluation of a stroke patient because it is available, fast, and non-sensitive to movement. CT can be helpful in ruling-out cerebellar hemorrhage in patients with acute vestibular syndrome, but hemorrhage is a rare cause of isolated vertigo and dizziness [83]. A posterior fossa ischemic stroke, which is more susceptible to manifest only as vertigo, is difficult to evaluate with CT and might be missed. Diffusion-weighted MRI (DWI) is an essential tool in detecting posterior circulation strokes due to its high sensitivity for acute ischemia. However, in the first 24 h, DWI may miss up to 15–20% of posterior fossa infarcts. Therefore, it should be interpreted alongside bedside exams and repeated imaging if clinical suspicion remains high [81,84,85,86,87].

The present study has limitations mainly on account of the fact it is not a systematic review or a meta-analysis. The heterogeneity of the vestibular disorders in the review did not allow a detailed analysis of each topic.

The differentiation between peripheral and central causes of acute vestibular syndrome (AVS) remains a critical concern in clinical practice. While MRI, particularly DWI, is widely regarded as the gold standard for identifying ischemic stroke, evidence has emerged that it may be insensitive in the early hours of posterior fossa infarction, missing up to 20% of cases in the first 24 h [84].

In contrast, the HINTS (Head-Impulse, Nystagmus, Test-of-Skew) examination, when performed by trained clinicians, has been shown in some studies to be more sensitive than early MRI in detecting central causes of AVS [84,85]. A positive HINTS result (normal head impulse, direction-changing nystagmus, or skew deviation) suggests a central lesion and may prompt expedited neuroimaging or neurology referral. These findings support the use of bedside examination as a first-line tool, particularly in settings where MRI access is limited or delayed. However, the HINTS exam also has limitations. Its accuracy is heavily operator dependent, and incorrect interpretation can lead to misdiagnosis. Additionally, the HINTS battery has limited utility outside the context of acute, continuous vertigo, and may not be reliable in episodic or positional vertigo. Some studies have also noted false-positive results in peripheral vestibular disorders and false negatives in small strokes. Therefore, while HINTS may complement imaging and, in some contexts, outperform early MRI for differentiation of central versus peripheral vertigo, it should be interpreted in the broader clinical context and ideally used in conjunction with imaging, not as a replacement. For a direct comparison of HINTS examination and MRI in evaluating AVS, please refer to Table 2. Clinicians must consider the patient’s risk factors, symptom duration, and the availability and timing of diagnostic tools to optimize outcomes [87]. Moreover, an often underrecognized phenomenon in clinical imaging is MRI-induced vertigo. Some patients undergoing MRI, particularly with high-field strength machines (e.g., 3 Tesla), may experience transient vertigo, dizziness, or nausea during or immediately after the scan. This phenomenon has been linked to the interaction between strong static magnetic fields and the vestibular apparatus, particularly through the stimulation of endolymphatic fluid movement in the semicircular canals. Lorentz forces generated by the movement of electrolytes in the inner ear can cause false signals interpreted by the brain as motion, leading to vertiginous sensations. The symptoms are typically short-lived and resolve spontaneously after removal from the magnetic field. However, they may cause discomfort or confusion, particularly in patients already undergoing evaluation for vestibular symptoms. It is important for clinicians to recognize and differentiate MRI-induced vertigo from pathological findings, especially when symptoms are reported during the imaging process rather than preceding it [88,89].

While MRI remains a critical tool in the evaluation of vertigo, awareness of its potential to induce vestibular symptoms helps avoid misinterpretation and enhances patient counseling prior to scanning.

### 3.2. Other Imaging Modalities (Ultrasound and Functional Imaging)

In addition to computed tomography (CT) and MRI, other imaging techniques, such as ultrasound and functional neuroimaging, may contribute to the diagnostic process or understanding of vestibular disorders, particularly in specific clinical contexts or research environments.

Ultrasound, especially transcranial Doppler (TCD) sonography, is a non-invasive method that enables real-time assessment of cerebral hemodynamics. TCD can be useful in evaluating patients with suspected vertebrobasilar insufficiency—a potential central cause of vertigo—by assessing blood flow velocity in the posterior circulation. Some studies suggest that TCD may identify flow reductions or vasospasm in the vertebral or basilar arteries in patients presenting with dizziness or vertigo. However, its clinical utility is limited by operator dependency, difficulty in insonating posterior circulation in certain patients, and relatively low sensitivity and specificity. Despite these limitations, ultrasound remains a cost-effective, accessible adjunct tool, particularly in emergency or bedside evaluations where vascular involvement is suspected [90].

Functional imaging techniques, including functional MRI (fMRI) and positron emission tomography (PET), are primarily utilized in research to study the neural correlates of vestibular processing. fMRI has been instrumental in identifying activation patterns in cortical and subcortical regions involved in balance and spatial orientation, such as the parietal operculum, insula, cerebellum, and vestibular cortex [91]. Similarly, PET imaging has been used to assess regional cerebral blood flow or metabolic activity during vestibular stimulation or in patients with chronic dizziness syndromes. These approaches have contributed to our understanding of central compensation mechanisms and perceptual disturbances in vestibular disorders [92].

Though these modalities are not part of routine diagnostic workflows in current clinical practice, acknowledging them within the broader context of vestibular imaging is important. They offer valuable insights into the central integration of vestibular input and may play a greater role in the future, especially as technology advances and functional-imaging techniques become more widely accessible in clinical settings.

### 3.3. Imaging Resolution Limitations and Lesion Detectability

The detectability of lesions in peripheral vestibular disorders is critically limited by the spatial resolution of imaging modalities and the size of the target anatomical structures. For example, in vestibular neuritis, the inflammation typically affects the superior or inferior vestibular nerve, which has an approximate diameter of 0.5–1.5 mm. Similarly, structures like the saccule and utricle involved in Meniere’s disease or the membranous labyrinth have dimensions in the range of a few millimeters. In contrast, acoustic neuromas can range from a few millimeters (e.g., 2–5 mm intracanalicular tumors) to several centimeters if they grow into the cerebellopontine angle [93]. Clinically available MRI scanners typically operate at 1.5T or 3T field strengths, offering voxel resolutions in the range of 0.5–1.0 mm isotropic in high-resolution inner ear protocols. CT, particularly high-resolution temporal bone CT, can achieve slice thicknesses of 0.5 mm, which is sufficient to delineate bony abnormalities such as canal dehiscence, but insufficient for resolving soft tissue structures like the vestibular nerve. Given these constraints, small lesions or pathological changes below the spatial resolution threshold—such as displaced otoconia in BPPV or early-stage neuritis—may not be visible on imaging. However, pathological changes such as inflammation, demyelination, or breakdown of the blood–labyrinth barrier can lead to altered MRI signal characteristics. For instance, the accumulation of contrast agent (gadolinium) in the perilymphatic space highlights endolymphatic hydrops in Meniere’s disease; similarly, enhancement of the vestibular nerve on post-contrast 3D FLAIR MRI sequences can suggest neuritis [94]. In such cases, the change in signal intensity or contrast uptake due to increased vascular permeability or tissue edema enables indirect detection of otherwise microscopic lesions. This emphasizes the diagnostic value of delayed contrast-enhanced MRI sequences in identifying inflammatory lesions and differentiating them from central causes of vertigo, even if the lesion itself is not directly resolved in detail. Furthermore, ongoing advances in image acquisition techniques, coil technology, and artificial intelligence-driven image reconstruction may push the resolution limits closer to the micrometer scale, allowing future detection of currently invisible pathological changes [4,6,18,29,30]. Emerging evidence suggests that sex-specific variability in interoceptive awareness may influence the perception, reporting, and clinical evaluation of vestibular symptoms. Studies indicate that females may report dizziness and vertigo more frequently and with greater severity, potentially due to heightened interoceptive sensitivity and hormonal influences on vestibular processing [95].

## 4. Conclusions

Dizziness and vertigo are frequent symptoms in clinical practice, often requiring careful distinction between peripheral and central vestibular causes. Diagnosis relies primarily on clinical evaluation and functional vestibular tests, with imaging—especially MRI—serving as a valuable tool in selected cases.

Each vestibular disorder has a unique diagnostic profile. Imaging is essential for identifying conditions like acoustic neuroma or superior canal dehiscence, while in other conditions, such as BPPV or vestibular neuritis, it plays a secondary, confirmatory role. Advances in imaging, particularly high-resolution CT and specialized MRI sequences, are enhancing diagnostic capabilities, especially in visualizing subtle inner ear pathologies.

Looking forward, artificial intelligence [92] offers promising potential to improve the sensitivity and interpretation of imaging for vestibular disorders. By integrating these technologies with established clinical workflows, diagnostic accuracy and patient outcomes may be further improved.

## Figures and Tables

**Table 1:** Common vestibular disorders, key symptoms, diagnostic methods, and the role of imaging in their diagnosis.

Vestibular Disorder	Primary Symptoms	Diagnostic Methods	Imaging Role
Vestibular neuritis	Vertigo, imbalance, nausea	Clinical examination, VNG	MRI (for differential diagnosis)
Meniere’s disease	Vertigo, hearing loss, tinnitus	Clinical examination, audiometry	MRI (for hydrops)
BPPV	Positional vertigo	Dix–Hallpike maneuver	Not typically required
Acoustic neuroma (vestibular schwannoma)	Unilateral hearing loss, tinnitus, imbalance	Audiometry, VNG, MRI	MRI (gold standard), essential for diagnosis
Superior canal dehiscence	Autophony, sound intolerance, vertigo	Audiometry, VEMP, CT	CT for confirmation, MRI (optional)

**Table 2:** Comparison of HINTS examination and MRI in evaluating acute vestibular syndrome (AVS).

Feature	HINTS Examination	MRI (DWI Sequence)
Purpose	Bedside test to differentiate central vs. peripheral AVS	Imaging to detect structural CNS lesions (e.g., stroke)
Sensitivity for stroke (early)	High (approaching 100% in expert hands) [83]	Lower in early phase (misses up to 20% of posterior strokes) [82]
Specificity	High when correctly performed	High
Time to result	Immediate (bedside)	Delayed (requires scanner availability)
Availability	Requires clinical expertise, no equipment	Dependent on MRI access
Limitations	Operator dependent, limited use in episodic vertigo	Limited sensitivity in hyperacute stroke, not feasible in all settings
Best use scenario	Emergency room, acute continuous vertigo	Confirmation of suspected central lesion, atypical presentation
Complementarity	Should be used first in AVS, may guide need for MRI	Ideal as a follow-up when central cause is suspected or HINTS is inconclusive
